# Parameters Associated with Significant Liver Histological Changes in Patients with Chronic Hepatitis B

**DOI:** 10.1155/2014/913890

**Published:** 2014-01-28

**Authors:** Li Xiao, Jianchun Xian, Yang Li, Aiwen Geng, Xiuzhen Yang, Libin Han, Hongtao Xu

**Affiliations:** Department of Liver Diseases, Taizhou People's Hospital, Jiangsu 225300, China

## Abstract

This study aimed to evaluate factors associated with significant liver histological changes. Liver biopsies from 157 CHB patients were retrospectively analyzed. Only ALB was significantly correlated with advanced liver necroinflammatory (*P* = 0.001). Age, ALB, GLOB, AST, PLT, and PT were independent predictors of significant fibrosis (*P* = 0.002, *P* < 0.001, *P* = 0.001, *P* = 0.048, *P* < 0.001, and *P* = 0.001, resp.). AST, WBC, and HBV DNA were significantly correlated with advanced fibrosis in normal ALT patients (*P* < 0.001, *P* = 0.041, and *P* = 0.012, resp.) and age, ALB, GLOB, PLT, and PT in patients with abnormal ALT (*P* = 0.003, *P* < 0.001, *P* = 0.004, *P* < 0.001, and *P* = 0.002, resp.). Age, AST, GGT, PLT, and PT were significantly associated with advanced fibrosis in HBeAg+ patients (*P* = 0.01, *P* = 0.016, *P* = 0.027, *P* = 0.016, and *P* = 0.009, resp.) and ALB, GLOB, WBC, PLT, and PT in HBeAg− patients (*P* < 0.001, *P* = 0.004, *P* = 0.005, *P* < 0.001, and *P* = 0.035, resp.). PLT was an excellent predictor for cirrhosis (*P* < 0.001 and AUROC = 0.805). ALT was not predictive of advanced fibrosis for patients with HBeAg+ or HBeAg− (*P* = 0.273 and *P* = 0.599, resp.). PLT was an excellent predictor for cirrhosis in CHB patients. Liver histopathology can be recommended for chronic HBV carriers of older age, with normal ALT, lower PLT, and lower ALB.

## 1. Introduction

Globally, there are approximately 350–400 million people infected with chronic hepatitis B virus (CHB) [1] and in China, a high endemic area, there an estimated 93 million people infected [2]. Without treatment, 15 to 40% of people with chronic HBV develop cirrhosis with a risk of developing hepatocellular carcinoma (HCC) [3, 4]. The clinical course of chronic HBV infection ranges from an inactive carrier state to cirrhosis, hepatic decompensation, and HCC [5–7]. Fibrosis often evolves insidiously, especially in inactive HBV carriers [8]. Active inflammation appears to be the driving force for development of fibrosis [9].

Liver biopsy remains the investigation of choice for assessment of inflammation and fibrosis. Liver biopsy is recommended for certain patients with chronic HBV infection especially older patients, those with persistent HBV DNA levels above 2,000 IU/mL and ALT 1-2 × the upper limit of normal (ULN), according to the Asian Pacific Association for the Study of the Liver (APASL), the European Association for the Study of the Liver (EASL), and the American Association for the Study of Liver Diseases (AASLD) [10–12]. Antiviral treatment is recommended when liver histology reveals moderate-to-severe active necroinflammation and/or significant fibrosis by METAVIR scoring [10–12]. Although liver biopsy is often essential in the management of patients with liver disease, physicians and patients are concerned about the invasive nature of the procedure and potential complications [13–15]. Sampling error and intraobserver variations are also not infrequent [16, 17]. Therefore, the development of noninvasive markers for significant liver disease is an attractive supplementary tool. Some noninvasive methods are used to assess liver fibrosis, such as transient elastography (TE) (FibroScan), FibroTest-ActiTest (Biopredictive, Labcorp) [14], and Fibrospect II (Prometheus) [9]. However, noninvasive methods are expensive and/or require equipment that is not widely available. These predictive markers of advanced liver pathology are useful screening tools that complement liver biopsy or help identify high-risk patients who are unwilling to undergo a liver biopsy. Several recent studies reported a relationship between liver function tests, HBV DNA levels, clinical characteristics, and other parameters with liver fibrosis [18–22]. However, these studies were limited by either a small number of parameters or only contained specific groups of patients, for example, with HBeAg+ and HBeAg− or normal and abnormal ALT. To fully analyze the relationship between these parameters and liver histology in different patient groups, we retrospectively analyzed HBeAg status, ALT levels, age, and HBV DNA from 157 patients with chronic HBV infection who underwent liver biopsy from Oct 2009 to Dec 2012 at Taizhou People's Hospital, Jiangsu, China.

## 2. Methods

### 2.1. Patients

Entry criteria to the study included treatment-naïve patients with chronic HBV infection who had a liver biopsy between Oct 2009 and Dec 2012 at the Taizhou People's Hospital, Jiangsu, China. Patients were recruited if they were positive for hepatitis B surface antigen (HBsAg) for at least 6 months, with an HBV DNA level of more than 500 copies/mL and a prothrombin time of less than 18 sec. We excluded patients (1) with viral coinfections, including HCV, HDV, and HIV infection, (2) with decompensated liver disease, (3) with metabolic or autoimmune liver disease, and (4) with a history of hepatotoxic drug ingestion and a high alcohol intake (20 grams per day for female, 30 grams per day for male). Written consent was obtained prior to liver biopsy.

Fasting serum samples were tested one day prior to liver biopsy for laboratory parameters by standard methods. Fifteen parameters including clinical, biochemical, and hematological variables were analyzed: age, gender, HBeAg status, HBV DNA level, PT (prothrombin time), ALB (albumin), GLOB (globulin), TB (total bilirubin), TP (total protein), ALT (alanine aminotransferase), AST (aspartate aminotransferase), ALP (alkaline phosphatase), GGT (glutamyl transferase), PLT (platelet count), and WBC (white blood cell). Hepatitis B serology including HBsAg/Ab, HBeAg/Ab, and HBcAb was detected by enzyme-linked immunosorbent assay (Fosun Pharmaceutical Co., Shanghai, China). HBV DNA level was determined with a lower limit of quantification of 500 copies/mL (about 100 IU/mL) by real-time polymerase chain reaction (PCR) (Fosun Pharmaceutical Co., Shanghai, China).

The main equipments used in this study included ABI Real-time PCR analyzer 7500 and BioTek Synergy2 Multi-Mode Microplate Reader, USA, HITACHI Biochemical analyzer 7600, and automatic blood analyzer Sysmex XT-2000i, Japan.

### 2.2. Liver Biopsy

Ultrasonographic-guided liver biopsies were performed using 16 G disposable needles (TSK corporation, Japan). Histological grading of necroinflammation and staging of liver fibrosis were considered reliable when the liver specimen length was ≥15 mm or the portal tract number ≥10 [23]. The histological diagnosis was established using hematoxylin and eosin (H&E) staining and Masson's trichrome stains of formalin fixed paraffin-embedded liver tissue. Two highly experienced liver pathologists reviewed all the liver specimens blinded to the patients' laboratory data. Histopathological findings were assessed and scored according to the METAVIR scoring system: A0 = no activity, A1 = mild activity, A2 = moderate activity, and A3 = severe activity; F0 = no fibrosis, F1 = portal fibrosis without septa, F2 = portal fibrosis with rare septa, F3 = numerous septa without cirrhosis, and F4 = cirrhosis [24].

### 2.3. Statistical Analysis

The baseline data of patients were presented as the median. Statistical analysis was carried out with SPSS 13.5 software for windows (SPSS Inc., Chicago, IL, USA). Chi-square test was used for categorical variables, the Student *t*-test for numerical variables, and logistic regression analysis to further test whether the identified variables associated with advanced histological abnormalities were independent risk factors. Spearman's rank correlation was used to assess correlation between variables, liver necroinflammation grades, and liver fibrosis stages. All *P* values were two sided and considered as statistically significant if <0.05.

## 3. Results

### 3.1. Patient Data

A total of 157 patients with all 15 clinical parameters available were enrolled into the study. There were 48 patients with inflammatory active grade A0-A1 and 109 at A2-A3 and 81 patients at liver fibrosis stage F0-F1 and 76 at F2–F4. The characteristics of all 157 patients are shown in Table 1.

**Figure 1:**
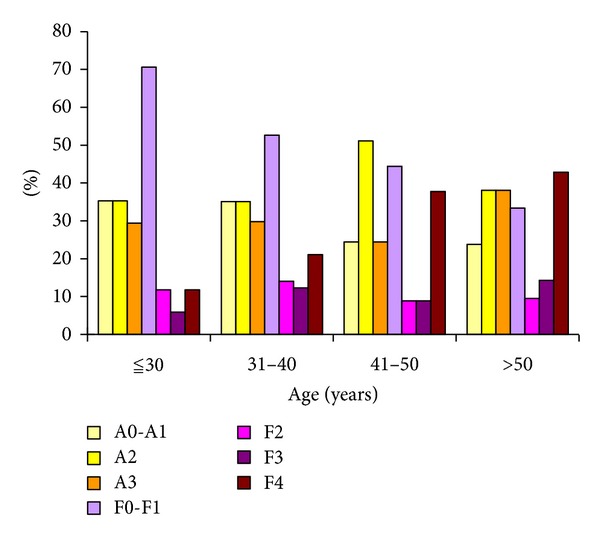
Distribution of liver necroinflammation and liver fibrosis (based on Metair Score) among different age groups in 157 patients with chronic HBV infection.

A total of 34 patients were aged ≦30, 57 aged 30–40, 45 aged 40–50, and 21 aged >50. There was no significant difference in necroinflammation grades among different age groups (*P* = 0.120); however, increasing age was independently associated with significant fibrosis (*P* = 0.034) (Figure 1).

**Figure 2:**
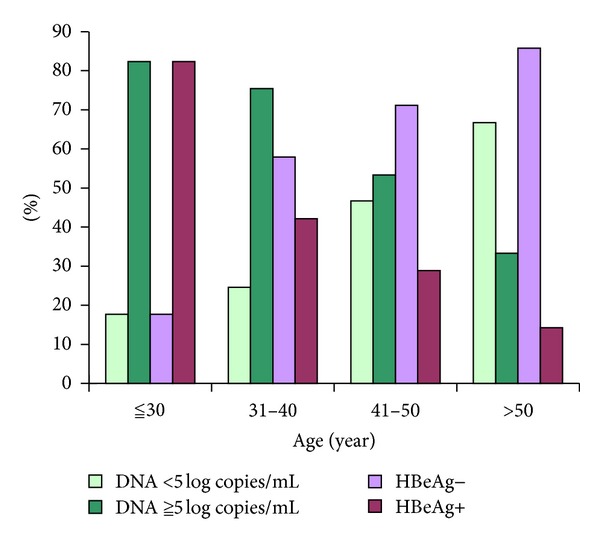
Characteristic of HBV DNA and HBeAg status among different age groups in 157 patients with chronic HBV infection.

The presence of HBeAg and hepatitis B viral load decreased with age: 28 (82%) and 28 (82%) had HBV DNA ≧ 5log copies/mL and HBeAg+, respectively, among the 34 patients aged ≦30. Seven (33%) and 3 (14%) had HBV DNA ≧ 5log copies/mL and HBeAg+, respectively, among the 21 patients aged >50 (Figure 2). The presence of HBeAg was associated with higher HBV DNA (*P* < 0.001).

**Figure 3:**
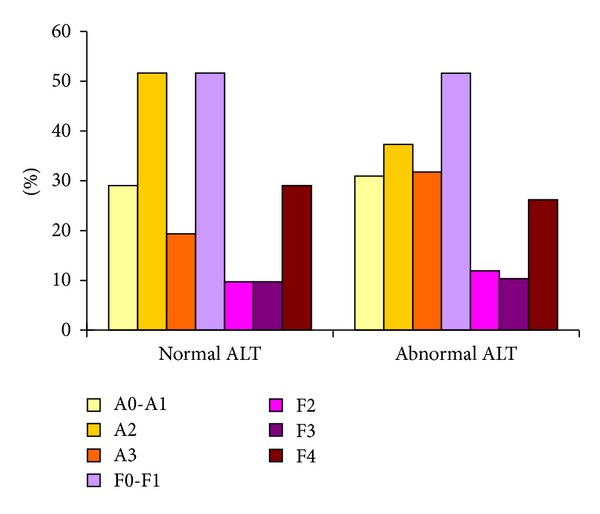
The distribution of liver necroinflammation and fibrosis in patients with normal and abnormal ALT.

A total of 31 patients (19.7%) had ALT levels within the normal range suggested by Prati et al. [25] (i.e., 30 U/L for men and 19 U/L for women) and 126 patients exceeded the Prati criteria. Nine of 31 (29%) patients with normal ALT had cirrhosis and 33 of 126 (26%) patients with abnormal ALT had cirrhosis. There was no significant difference in liver necroinflammation grades or fibrosis stages between patients with normal and abnormal ALT (*P* = 0.835 and *P* = 0.998, resp.) (Figure 3). Spearman's rank correlation showed that AST, WBC, and DNA were significantly correlated with advanced fibrosis in patients with normal ALT (*P* < 0.001, *P* = 0.041, and *P* = 0.012, resp.), while age, ALB, GLOB, PLT, and PT were significantly correlated with advanced fibrosis in patients with abnormal ALT (*P* = 0.003, *P* < 0.001, *P* = 0.004, *P* < 0.001, and *P* = 0.002, resp.).

**Figure 4:**
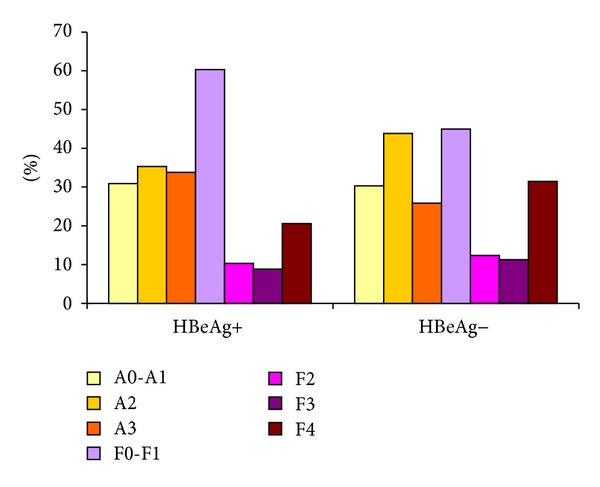
Distribution of liver necroinflammation and liver fibrosis (based on Metair Score) among HBeAg+ and HBeAg− groups.

Advanced necroinflammation was found in 39.8% of HBeAg+ patients and 55.1% of HBeAg− patients. Advanced fibrosis was found in 62.2% of HBeAg+ patients and in 69.7% of HBeAg− patients. There was no significant difference between HBeAg+ and HBeAg− groups in necroinflammation grades and fibrosis stages (*P* = 0.057 and *P* = 0.941, resp.) (Figure 4). Age, AST, GGT, PLT, and PT were significantly correlated with advanced fibrosis in HBeAg+ patients (*P* = 0.01, *P* = 0.016, *P* = 0.027, *P* = 0.016, and *P* = 0.009, resp.). ALB, GLOB, WBC, PLT, and PT were significantly correlated with advanced fibrosis in HBeAg− patients (*P* < 0.001, *P* = 0.004, *P* = 0.005, *P* < 0.001, and *P* = 0.035, resp.). There was no significant difference in fibrosis stages in patients with normal and abnormal ALT for both HBeAg+ and HBeAg− groups (*P* = 0.273 and *P* = 0.599, resp.).

### 3.2. Identification of Variables Predicting Advanced Liver Necroinflammation and Fibrosis

Two levels of liver fibrosis (F0-F1 and F2–F4) and liver necroinflammation (A0-A1 and A2-A3) were analyzed in 157 patients with chronic HBV infection. Of the 15 variables, only ALB was significantly correlated with advanced liver necroinflammation (OR = 0.888  *P* = 0.002). Spearman's rank correlation analysis showed that 10 variables (age, TB, ALB, GLOB, AST, ALP, GGT, WBC, PLT, and PT) were correlated significantly with advanced liver fibrosis (correlation coefficients were 0.245, 0.213, −0.291, 0.249, 0.235, 0.223, 0.288, −0.258, −0.383, and 0.256, resp.; *P* values were 0.002, 0.007, <0.001, 0.002, 0.003, 0.005, <0.001, 0.001, <0.001, and 0.001 resp.). Univariate analysis and logistic regression analysis revealed that only age, ALB, GLOB, AST, PLT, and PT were independent predictive factors and were significantly different in mild and moderate/severe fibrosis (Table 2). A lower ALB was independently associated with significant necroinflammation. Older age, higher AST, longer PT, lower PLT, and lower ALB were independently associated with significant fibrosis.

The area under ROC curve (AUC) of ALB for significant necroinflammation in all patients was 0.614. The 95% confidence interval was 0.501 to 0.727. PLT was a good predictor for cirrhosis and its AUROC was 0.805. The AUC of the predictors for cirrhosis was shown in Table 3.

## 4. Discussion

The development of noninvasive markers for liver fibrosis is an attractive option because of the associated risks of liver biopsy. Previous studies have shown inconsistent results and the optimal predictor for significant liver fibrosis is not known. This study investigated the association of various routinely available clinical parameters with liver histology.

Current guidelines for antiviral treatment of chronic HBV infection recommend therapy when ALT levels are more than twice the upper limit of normal; however, previous studies have not demonstrated a correlation between ALT levels and liver fibrosis [26–29]. We investigated the association between ALT, liver fibrosis, and necroinflammation and consistent with previous studies, found no significant correlation between ALT, liver fibrosis, necroinflammation, or with HBe status. We found that 29% of patients with normal ALT levels, using the reference ranges suggested by Prati et al. [25], had significant fibrosis. Therefore, using the ALT threshold of more than 2 × ULN, current guidelines may deny patients treatment, who have significant fibrosis or cirrhosis.

We found no significant association between HBeAg status and liver fibrosis. HBeAg status was, however, associated with higher HBV DNA levels and the presence of HBeAg and hepatitis B viral load decreased with age. The correlation between HBV DNA levels and liver fibrosis is controversial. Croagh et al. reported that HBV DNA was an independent predictor for significant fibrosis in HBeAg− but not HBeAg+ patients [20]. Seto et al. reported that HBV DNA levels had no correlation with liver histology, but the majority of the study population contained HBeAg+ patients [30]. Our study did not show any association between HBV DNA and liver histology in patients testing HBeAg+ nor HBeAg−. However, HBV DNA was significantly correlated with advanced fibrosis in patients with normal ALT. This may be explained by different ethnic groups, patient sample differences, HBV genotypes, and transmission route of HBV infection. Chinese mainland patients acquire the infection perinatally with liver injury starting early in life and the HBV genotypes are mainly B and C [31].

Most previous studies have focused on predictors for liver fibrosis and the main driving force for the development of fibrosis appears to be active inflammation. In the present study, we examined routine parameters for predicting liver necroinflammatory disease. Our study showed that only ALB among the 15 variables was negatively correlated with advanced liver necroinflammatory disease. Age, ALB, AST, PLT, and PT were independently associated with significant fibrosis in different chronic HBV groups, that is, HBeAg− and HBeAg+ patients [21, 26, 32–35]. Age is an important predictor and reflects progression of fibrosis in a time-dependent manner. Our study found age, ALB, GLOB, AST, PLT, and PT were associated with significant fibrosis in all patients with chronic HBV infection. AST had the lowest association with fibrosis, whereas PLT was an excellent predictor for significant fibrosis. A low platelet count is associated with advanced liver fibrosis through the altered production of thrombopoietin and is independent of demographic and biochemical characteristics, hepatic necroinflammatory activity, portal hypertension, and splenomegaly [36].

There are limitations of this study. HBV genotyping was not performed, which may have affected the results as previous small-scale studies found genotype C was associated with significant histological abnormalities [33, 37].

In conclusion, ALT is a poor marker when considering antiviral therapy because of its poor correlation with significant liver injury in patients with chronic HBV infection. Lower levels of PLT were independently associated with significant fibrosis. If a liver biopsy is considered to assess disease activity and fibrosis, it can be recommended for patients with chronic HBV infection, particularly for an older age group and patients with normal ALT and lower PLT and ALB.

## Figures and Tables

**Table 1:** Characteristics of 157 patients included in the study.

Variables	Total	A0-A1	A2-A3	F0-F1	F2–F4
Number of patients	157	48	109	81	76
Age (years)	38 (14–68)	36 (14–58)	39 (18–68)	36 (14–64)	41 (25–68)
Gender (male)	136 (87%)	40 (83%)	96 (91%)	68 (84%)	68 (89%)
HBeAg+	68 (43%)	21 (44%)	47 (43%)	41 (51%)	27 (36%)
HBeAg−	89 (57%)	27 (56%)	62 (57%)	40 (49%)	49 (64%)
HBV DNA (log copies/mL)	6 (2–9)	5.5 (3–9)	6 (2–9)	6 (2–9)	5.5 (2–8)
PT (sec)	13 (8.2–17.8)	13 (8.2–16)	13.1 (9.2–17.8)	12.8 (8.2–16.7)	13.7 (9.5–17.8)
ALB (g/L)	42.5 (30.1–53.9)	43.3 (33.5–53.7)	42 (30.1–53.9)	43.3 (33.3–53.9)	41.15 (30.1–48.9)
GLOB (g/L)	31.2 (21.9–48.2)	30.5 (23.3–48.1)	31.9 (21.9–48.2)	30.2 (21.9–45.9)	33.1 (23.9–48.2)
TB (umol/L)	16.2 (5.9–218)	14.5 (6.1–84.7)	16.3 (5.9–218)	14.3 (5.9–218)	18.1 (6.1–170.5)
TP (g/L)	74.1 (59.5–92.5)	75.3 (64.4–92.5)	74 (59.5–87.1)	74 (60.6–92.5)	74.6 (59.5–89.3)
ALT (IU/L)	69 (13–1387)	64 (18–1387)	72 (13–1215)	62 (13–1387)	71 (17–999)
AST (IU/L)	46 (14–723)	44.5 (16–658)	47 (14–723)	40 (14–571)	58 (17–723)
ALP (IU/L)	79 (35–323)	73 (35–323)	80 (41–258)	74 (35–323)	88 (42–258)
GGT (IU/L)	43 (7–476)	38 (7–467)	46 (11–402)	30 (7–476)	62 (15–402)
PLT (×10^9^/L)	115 (19–314)	122.5 (43–257)	114 (19–314)	132 (28–257)	97 (19–314)
WBC (×10^9^/L)	4.69 (1.96–7.77)	4.68 (2.73–7.64)	4.7 (1.96–7.77)	5.11 (2.4–7.77)	4.32 (1.96–7.64)

Continuous variables are expressed as the median (range).

**Table 2:** Clinical parameters predictive of advanced liver necroinflammation and fibrosis.

		*t* test		Spearman's correlation		Logistic regression			
		*t* value	*P* value	Correlation coefficient	*P* value	OR value	*P* value	95% CI for OR	
								Lower	Upper
A2-A3	ALB	3.238	0.001	−0.236	0.003	0.888	0.002	0.823	0.958
F2–F4	Age	−3.075	0.002	0.245	0.002	2.113	0.002	1.330	3.356
	ALB	4.099	<0.001	−0.291	<0.001	0.119	0.037	0.016	0.879
	GLOB	−3.282	0.001	0.249	0.002	0.136	0.049	0.019	0.988
	AST	−1.997	0.048	0.235	0.003	1.031	0.011	1.007	1.055
	PLT	4.548	<0.001	−0.383	<0.001	0.991	0.037	0.982	0.999
	PT	−3.355	0.001	0.256	0.001	1.641	<0.001	1.252	2.151

**Table 3:** The area under ROC curve (AUC) of the identified variables for cirrhosis in all patients.

	AUC for cirrhosis	95% confidence interval	
		Lower	Upper
Age	0.728	0.610	0.846
ALB	0.721	0.600	0.841
GLOB	0.760	0.645	0.875
AST	0.681	0.554	0.808
PLT	0.805	0.702	0.908
PT	0.708	0.581	0.834
